# *In Vitro* Investigation of Heat Transfer Phenomenon in Human Immature Teeth

**DOI:** 10.5681/joddd.2014.039

**Published:** 2014-12-03

**Authors:** Maryam Talebi, Sahar Moghimi, Mina Shafagh, Hadi Kalani, Fatemeh Mazhari

**Affiliations:** ^1^Associate Professor, Dental Materials Research Center, School of Dentistry, Mashhad University of Medical Sciences, Mashhad, Iran; ^2^Assistant Professor, Department of Electrical Engineering, Ferdowsi University of Mashhad, Mashhad, Iran; ^3^MD, Department of Pediatric Dentistry, School of Dentistry, Mashhad University of Medical Sciences, Mashhad, Iran; ^4^PhD Candidate, Department of Mechanical Engineering, Ferdowsi University of Mashhad, Mashhad, Iran

**Keywords:** Finite element analysis, polymerization, pulp, thermal conductivity

## Abstract

***Background and aims.*** Heat generated within tooth during clinical dentistry can cause thermally induced damage to hard and soft components of the tooth (enamel, dentin and pulp). Geometrical characteristics of immature teeth are different from those of mature teeth. The purpose of this experimental and theoretical study was to investigate thermal changes in immature permanent teeth during the use of LED light-curing units (LCU).

***Materials and methods.*** This study was performed on the second mandibular premolars. This experimental investiga-tion was carried out for recording temperature variations of different sites of tooth and two dimensional finite element models were used for heat transfer phenomenon in immature teeth. Sensitivity analysis and local tests were included in the model validation phase.

*** Results.*** Overall, thermal stimulation for 30 seconds with a low-intensity LED LCU increased the temperature from 28°C to 38°C in IIT (intact immature tooth) and PIT (cavity-prepared immature tooth). When a high-intensity LED LCU was used, tooth temperature increased from 28°C to 48°C. The results of the experimental tests and mathematical modeling illustrated that using LED LCU on immature teeth did not have any detrimental effect on the pulp temperature.

*** Conclusion. ***Using LED LCU in immature teeth had no effect on pulp temperature in this study. Sensitivity analysis showed that variations of heat conductivity might affect heat transfer in immature teeth; therefore, further studies are required to determine thermal conductivity of immature teeth.

## Introduction

A number of products and procedures used in dental diagnosis and treatment, such as cavity preparation, light-curing of dental restorations, rotary instruments and high-intensity lasers, can exert harmful thermal effects on dental tissues. In addition, heat transfer in a tooth can be affected by factors such as the geometry of tooth components, thickness of enamel and dentin, type of restorative materials, blood perfusion and pulp circulation.^1-5^

There are very strict limitations on in vivo studies due to health care regulations imposed on human teeth and for this reason most experiments are carried out in vitro.^1^The investigation of heat transfer in tooth requires both experimental measurements and mathematical modeling.^6-17^ Experimental measurements are required to check the validity of modeling predictions. In mathematical modeling the structure of tooth is generally a simplified non-geometrical model containing only enamel, dentin and dental restorative materials. Unfortunately, these simplifications lead to multiple limitations. For instance, variation in tooth properties, different donors (age, sex and races), pulpal blood and dentinal fluid flow cannot be applied to the model.^1^

However, considering the variations in geometry as a result of changes in some properties (e.g. age) may provide a better comprehension of the process under study. Geometrical characteristics of immature teeth are different from those of mature teeth. Immature teeth, in contrast to mature ones, have open apices, lower dentin thickness, extended pulp area and dentinal tubules.^1^

The purpose of this in vitro study was to investigate thermal changes in different layers of immature permanent teeth during the use of LED LCU with different intensities in order to detect possible harmful effects.

## Materials and Methods

This in vitro study was performed on four second mandibular premolars (one pair of mature teeth and one pair of immature teeth) that were extracted for orthodontic therapy.

### Experimental Setup

General description: Two immature (intact and cavity-prepared) teeth were selected and the experimental setup consisted of three main parts.

- Heat generating and mouth cavity unit: The tooth was placed in a cast and inserted in a chamber filled with isothermal distilled water below the CEJ level. Water temperature was similar to that of tooth pulp and crevicular gingival fluid.
- Data acquisition, processing and visualization system.
- Light-curing unit: A light-emitting diode (Ivoclar Vivadent, Schaan, Liechtenstein) was used to generate heat.

**Figure 1.**
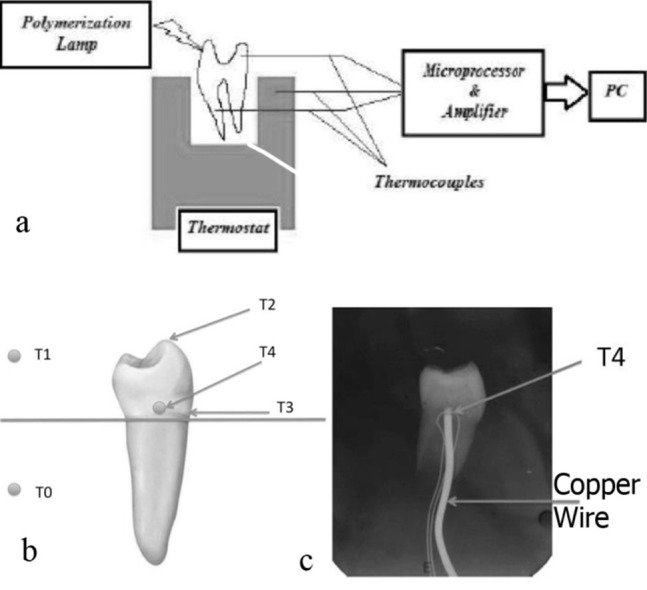
a) A schematic diagram of the experimental setup. b) Thermocouple locations in tooth: T0 (chamber of water); T1 (ambient air); T2 (top of the buccal cusp); T3 (cemento-enamel junction area above the water bath); T4 (inside the pulp). c) X-ray of thermocouple T4 and copper wire location.

A schematic diagram of the whole system is depicted in Figure 1a.

### Preparation Phase 

The intact immature tooth (IIT) was mounted on a thin, plastic plate and thermocouples were installed. Next, it was inserted into the wash bath. Thermocouples of type "K" and a wire with a diameter of 0.25 mm were applied and fixed by glue. The thermocouple wires were encased in Teflon tubes to prevent contact with each other. Because of the short diameter of the thermocouples they transferred a minimum amount of heat.

After the attachment of thermocouples a copper wire was inserted within the pulp chamber through the tooth root canal to simulate heat transfer at the dentin−pulp junction and pulp soft tissue. The teeth were radiographed after assembling the thermocouples and copper wire (Figure 1).

The thermocouple signals were amplified and filtered for noise reduction. A PC equipped with a serial port handled the measured signals. A graphical user interface (GUI) was utilized for real-time illustration of calibrated temperature values. A detailed description of the signal acquisition system is provided in a previous publication.^18^

### Experimental Procedure

At first the intact immature tooth was inserted into water and left for 30 minutes for equilibrium at water temperature (37°C). Then an LED LCU was used with low and high intensities (650 mW/cm ^2^and 1200 mw/cm^2^, respectively) at a 1-mm distance from the tooth for 30 seconds. The relaxation time of the temperature equilibration was about 30 minutes between experiments until the primary temperature of the tooth was restored.

In the modeling phase the effect of source heating was included by considering time variation boundary conditions of the buccal cusp. The time variation values were those recorded by T2 in Figure 1b.

**Figure 2.**
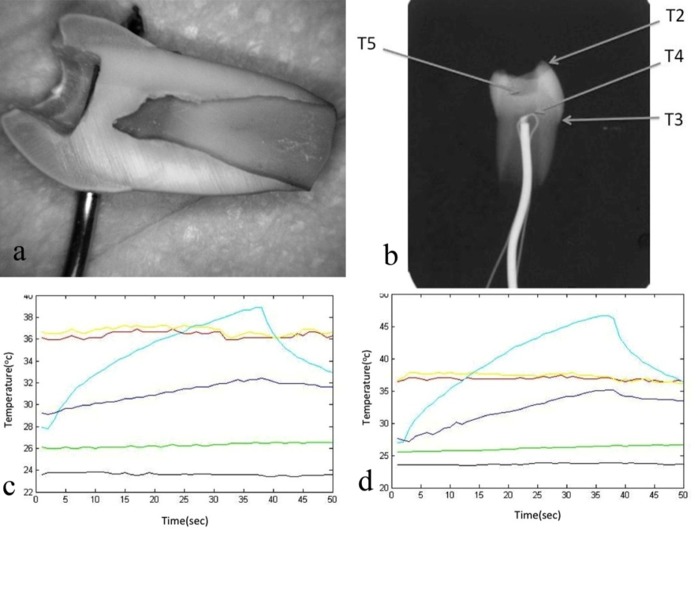
Cavity-prepared immature tooth: a) photography, b) radiography. Temperature changes recorded in cavity-prepared immature tooth during application of: c) low-intensity LED LCU; d) high-intensity LED LCU. Yellow: T0; black: T1; cyan: T2; green: T3; red: T4; blue: T5 (according to Figure 1.b).

After preparing a Cl I cavity in the occlusal surface with 0.5 mm of dentin remaining near the pulp area (checked by radiography, Figure 2b), the previous processes were repeated.

A similar cavity was prepared in the intact half of the thin tooth and photography was carried out under a stereomicroscope (Dino Lite AM9413T ×10, ×50, ×200, Taiwan) for mathematical modeling.

### Modeling of Heat Transfer in the Tooth

Transient modeling of heat transfer: Numerical simulation was carried out based on a two-dimensional finite element method (2D FEM). The model was designed based on the photographs taken from a longitudinal section of the tooth according to the symmetric structure of the second mandibular premolar. The 2D FEM modeling can be utilized for modeling 3D symmetrical objects for the sake of computational simplicity.^14^ After taking a photograph under a stereomicroscope, the outer layers of the teeth were determined using a professional CATIA software package. FEM analytical analysis was performed by ABAQUS 6.11 software package. Heat transfer was quantitatively investigated using 2D FEM. Thermal diffusivity and heat transfer in different layers of the teeth were determined continually.

Local test: For a more accurate assessment of the model, three points in enamel, dentin and pulp were selected in all the teeth and modeling conditions were set according to the experimental procedures.

Sensitivity analysis: Different values of thermophysical properties of human teeth are reported in the literature.^1,8,19^ In order to investigate the effect of changes in parameters, time-varying temperatures of the locations T3 and T4 were calculated using FEM for different values of thermophysical properties and compared to the values recorded by the sensors (Figure 1b).

## Results

### In Vitro Measurement of Heat Transfer in Teeth 

Overall, thermal stimulation for 30 seconds with LED LCU with at intensity increased the temperature from 28°C to 38°C (a 10°C increase) in IIT and PIT. When high-intensity LED LCU was used, tooth temperature increased from 28°C to 48°C (a 20°C increase) as shown in Figures 2c-2d.

CEJ area temperature was similar to the ambient air (24°C); however, when cavity preparation was carried out it increased to 27°C.

In all the above situations pulp temperature was similar to bath water without any changes (37°C). At the base of the IIT (T5), temperatures increased to 32°C (with low-intensity LED) and to 35°C (with high-intensity LED).

### Heat Modeling

Transient heat modeling was carried out according to the experimental procedures. The profile of temperature distribution was recorded at 5, 10, 15, 20, 25 and 30 seconds (considering the periods of LED application) and is illustrated for PIT in 3. The same process was repeated for the other teeth (IIT, IMT, and PMT=cavity-prepared mature tooth).

**Figure 3.**
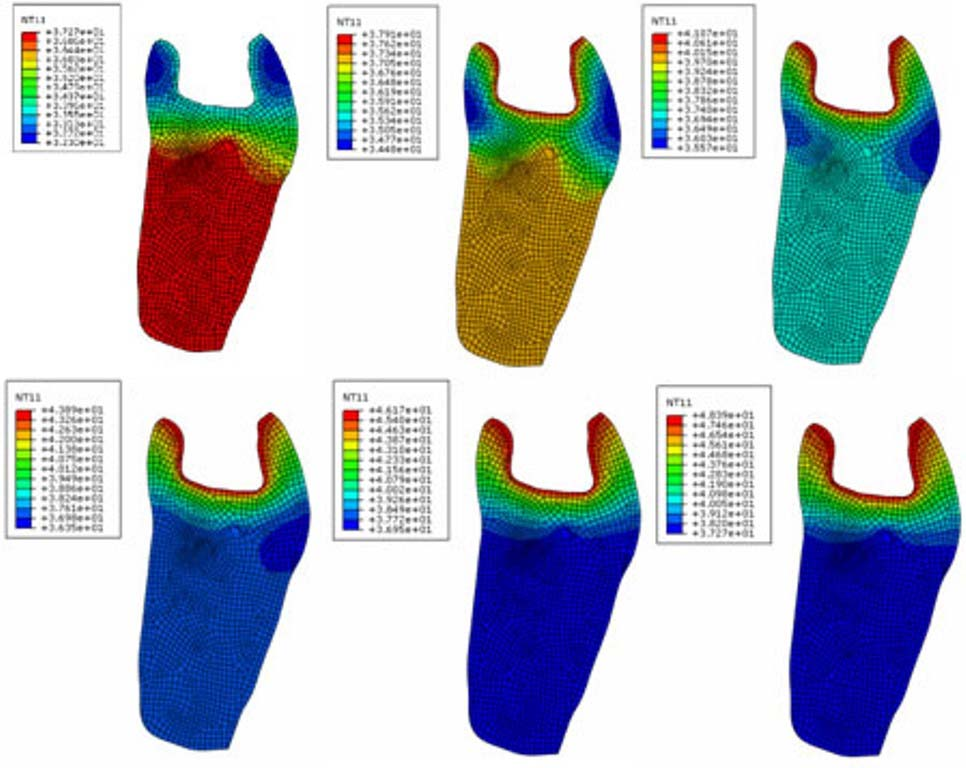
The results of transient heat modeling for 30 seconds of application of LED LCU with an intensity of 1200 mW/cm^2^ in PIT. From top left to bottom right: 5, 10, 15, 20, 25 and 30 seconds, respectively.

The results of the local tests by applying the high-intensity of LED to IIT are illustrated in 4. A comparative study of local tests is represented in Table 1.

**Figure 4.**
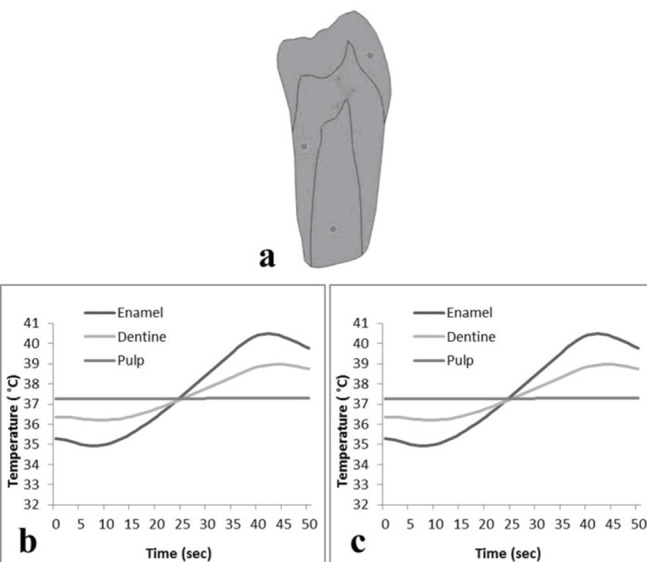
Local test results: a) Locations of local tests are denoted as red circles. Time-varying temperature in specified location in (a), as a result of application of the high intensity of LED LCU (1200 mW/cm^2^) to b) IIT and c) PIT.

**Table 1:** Local test by using high-intensity LED

Tooth	Temperature increase	Temperature increase
	(enamel)	(dentin
IIT	5.8°C (35.2–41)	2.5°C (36.5–39)
PIT	10.8°C (35.2–46)	5.5°C (36.5–42)
IMT	3.3°C (35.5–38.8)	1.2°C (36.6–37.8)
PMT	9.5°C (35.5–45)	3.4°C (36.6–40)

Sensitivity analysis tests were performed by applying changes to the enamel and dentin densities as well as enamel and dentin heat conductivity coefficients. Temperature changes at the locations T3 and T4 as a result of changes in enamel and dentin densities were negligible and therefore not illustrated in this report. The results of sensitivity analysis by applying changes in heat conductivity coefficients of enamel and dentin are shown in 5.

**Figure 5.**
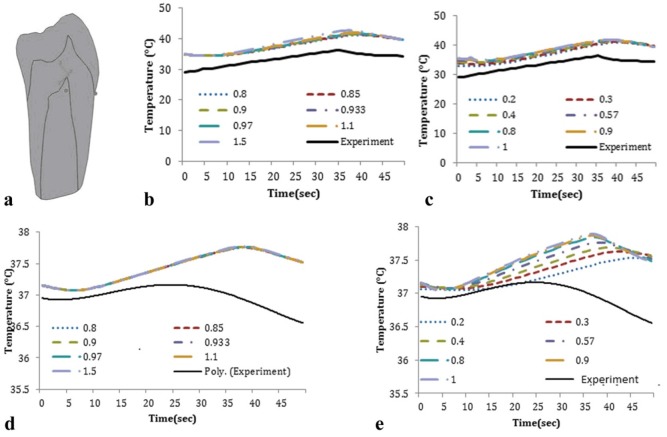
The results of sensitivity analysis. a) Locations of sensitivity analysis tests, corresponding to T3 and T4, respectively. Temperature variations as a result of changes in heat conductivity coefficients of enamel and dentin at locations T3, (b) and (c), and T4, (d) and (e).

## Discussion

Heat generated within the tooth during clinical dentistry can cause thermally induced damage to hard and soft components of the tooth (enamel, dentin and pulp). An intrapulpal temperature exceeding 42°C could result in irreversible pulp damage.^8,14^ All the previous investigations on heat transfer have been carried out on mature teeth.^1^However, considering the variations in tooth geometry that occur as a result of aging a better comprehension of the process under study can be provided because geometrical characteristics of immature teeth are different from those of mature teeth.^1^ In this study, heat transfer as a result of using LED LCU was investigated in immature teeth and then compared to mature ones. Similar to the strategy adapted by Preiskorn et al,^8^ in order to simulate the transfer of heat at the dentin‒pulp junction and soft pulp tissue, a copper wire was placed within the pulp chamber and its location was verified by radiography. Similar to Whilst Hanning et al^6^06 the pulp chamber was filled with warm water (37°C) to mimic heat transfer through the soft tissue in the pulp chamber. These in vitro measurements with simulated pulp tissue are more reliable than those carried out with an empty pulp chamber since they simulate the undeniable role of the pulp in heat transfer. In vitro experiments in IIT showed a 10°C and 20°C increase in temperature in T2. In the PIT these values were 4°C and 7°C. The difference can be attributed to geometrical changes due to cutting the enamel and dentin. In this situation the tooth with exposed dentin responded more quickly to thermal stimulation in comparison to the intact one. In both the teeth above, there was no thermal increase in pulp (37°C) with the use of LED LCU with the two intensities. Tunc et al also reported that long-time exposure to LED LCU did not result in a considerable rise in the temperature within a pulp chamber model.^14^

Unlike a previous study carried out by Preiskorn et al^8^ we decided to extend the relaxation time between the experiments to 30 minutes to allow the tooth to regain its primary temperature. By doing this we tried to bring the initial condition of each model closer to that of the other models, which would result in more meaningful comparisons

The initial condition for modeling was produced by considering the boundary temperature of the regions in the water bath at 37°C and then simulating the heat transfer phenomenon for 30 minutes to bring the initial condition closer to the tooth condition prior to extraction. A 5°C difference exists in the initial conditions of enamel recorded during the experiment and those obtained by modeling (Figures 2and4). The sensor T2 in Figure2 is located at the surface of tooth; therefore it mostly takes up the ambient air temperature (28°C). However, the initial condition in 4 is a result of heat conduction from water (at 37°C) through the copper wire (which is simulated for 30 minutes).

During IIT and IMT transient heat modeling, surface temperature increased by using an LED unit. Pulp temperature remained at 37°C under all the conditions. This shows that using LED did not produce harmful heat when used according to the manufacturers’ operating procedures in immature tooth.

For a more accurate study of the transient temperature, local tests were performed on three points in enamel, dentin and pulp by using a high-intensity LED light-curing unit. In PIT and PMT, the tooth responded more quickly to thermal stimulation (Table 1). This is due to the removal of enamel and as a result of direct heat transfer to dentin.

In this modeling of heat transfer all the thermophysical properties of immature and mature teeth were extracted from the literature. Since these parameters were chosen to be the same in the models of immature and mature teeth, all the differences in the temperature are due to geometrical characteristics. For example, an immature tooth has an expanded pulp area and dentinal tubules, open apex and less dentin diameter in comparison to a mature tooth.

This study was carried out based on the thermophysical properties found in the literature.^1,8,19^ To investigate the effect of changes in the tooth tissue properties on the obtained temperature, the model was simulated several times with different values for thermophysical properties.

Modeling results were similar to in vitro experiments (5). The small differences between experimental and modeling results in this study were related to different initial temperatures. In this study the difference between recorded temperatures and those obtained by modeling was smaller than what was previously reported by other researchers.^8^

Variations in the densities of enamel and dentin did not have an effect on the temperature in immature teeth (5). However, variations in the coefficient of heat conductivity in enamel and dentin caused changes in the profile of temperature rise in immature teeth.

In mathematical modeling the tooth is simplified as a system of geometrical areas containing dentin, enamel and dental materials. This simplification leads to approximate results. Moreover, even though the application of a 2D FEM can be justified due to the symmetric structure of the second mandibular premolars, this assumption may also result in small variations in the results from the actual values. Therefore, the measured and estimated values reported in this research cannot be directly assumed for temperature changes during in vivo studies.

Although in this study the effect of variations in model properties were investigated by performing sensitivity analysis, further studies are still required to determine accurate thermal properties of immature tooth.

In this study the structure of dentin and enamel were assumed to be homogenous in order to simplify the modeling procedure. More accurate results can be obtained by assuming a heterogeneous structure for the aforementioned tissues according to a reasonable statistical approach.

## Conclusion

Using LED LCU in immature teeth had no effect on pulp temperature in this study. Sensitivity analysis showed that variations of heat conductivity can affect heat transfer in immature teeth. Therefore, further studies are required to determine thermal conductivity of immature teeth.

## References

[1] Lin M, Xu F, Lu TJ, Bai BF (2010). A review of heat transfer in human tooth--experimental characterization and mathematical modeling. Dent Mater.

[2] Liang S, Sa Y, Jiang T, Ma X, Xing W, Wang Z (2013). In vitro evaluation of halogen light-activated vs chemically activated in-office bleaching systems. Acta Odontol Scand.

[3] Thomas MS, Kundabala M (2012). Pulp hyperthermia during tooth preparation: the effect of rotary instruments, lasers, ultrasonic devices, and airborne particle abrasion. J Calif Dent Assoc.

[4] Liang S, Sa Y, Sun L, Ma X, Wang Z, Xing W (2012). Effect of halogen light irradiation on hydrogen peroxide bleaching: an in vitro study. Aust Dent J.

[5] Davis S, Gluskin AH, Livingood PM, Chambers DW (2010). Analysis of temperature rise and the use of coolants in the dissipation of ultrasonic heat buildup during post removal. J Endod.

[6] Hannig M, Bott B (1999). In-vitro pulp chamber temperature rise during composite resin polymerization with various light-curing sources. Dent Mater.

[7] Linsuwanont P, Palamara JE, Messer HH (2007). An investigation of thermal stimulation in intact teeth. Arch Oral Biol.

[8] Preiskorn M, Zmuda S, Trykowski J (2003). In vitro investigations of the heat transfer phenomena in human tooth. Acta Bioeng Biomech.

[9] Linsuwanont P, Palamara JE, Messer HH (2008). Thermal transfer in extracted incisors during thermal pulp sensitivity testing. Int Endod J.

[10] Secilmis A, Bulbul M, Sari T, Usumez A (2013). Effects of different dentin thicknesses and air cooling on pulpal temperature rise during laser welding Lasers. Med Sci.

[11] Lipski M, Woźniak K, Lichota D, Nowicka A (2011). Root surface temperature rise of mandibular first molar during root canal filling with high-temperature thermo plasticized Gutta-Percha in the dog. Pol J Vet Sci.

[12] Onisor I, Asmussen E, Krejci I (2011). Temperature rise during photo-polymerization for onlay luting. Am J Dent.

[13] Raucci-Neto W, Pécora JD, Palma-Dibb RG (2012). Thermal effects and morphological aspects of human dentin surface irradiated with different frequencies of Er:YAG laser. Microsc Res Tech.

[14] Tunc EP (2007). Finite element analysis of heat generation from different light-polymerization sources during cementation of all-ceramic crowns. J Prosthet Dent.

[15] Ana PA, Velloso WF, Zezell DM (2008). Three-dimensional finite element thermal analysis of dental tissues irradiated with Er:YAG laser. Rev Sci Instrum.

[16] Jakubinek MB, O’Neill C, Felix C, Price RB, White MA (2008). Temperature excursions at the pulp–dentin junction during the curing of light-activated dental restorations. Dent Mater.

[17] Oskui IZ, Ashtiani MN, Hashemi A, Jafarzadeh H (2013). Thermal analysis of the intactmandibular premolar: a finite element analysis. Int Endod J.

[18] Saghlatoon H, Soleimani M, Moghimi S, Talebi M (2012). An experimental investigation about the heat transfer phenomenon in human teeth. ICEE.

[19] Brown WS, Dewey WA, Jacobs HR (1970). Thermal properties of teeth. J Dent Res.

